# Supplementary material for: Emergence of artemisinin-based combination therapy resistance markers in Plasmodium falciparum from the Brazilian tri-border region of the Guiana Shield
Source: Antimicrob Agents Chemother. 2026 Mar 12;70(4):e01525-25. doi: 10.1128/aac.01525-25 (PMC13041304; doi:10.1128/aac.01525-25)
Supplement: Supplemental material — Supplemental methods. [file aac.01525-25-s0001.pdf]

SUPPLEMENTARY MATERIAL

Supplement to: **Emergence of Artemisinin-based Combination Therapy Resistance Markers in *Plasmodium falciparum* from the Brazilian Tri-Border Region of the Guiana Shield**

Yanka E. A. R. Salazar, Antonio M. Rezende, Maria C. S. B. Puça, Jaime Louzada, Maria E. P. Mascarenhas, Sonja Lagström, Danielle Fletcher, Joseli Oliveira-Ferreira, José P. Gil, Tais N. de Sousa

METHODS

Study Site and Sample Characteristics

Sample collection was carried out in Boa Vista, the capital of Roraima state, and in Pacaraima, which is located on the border with Venezuela. Pacaraima is home to indigenous groups, including the Makuxi, Taurepang, and Wapixana. This region borders Venezuela and Guyana, and is near French Guiana, all areas characterized by high population mobility, particularly among informal gold miners operating in remote forested areas (1, 2).

The analysis included 99 *P. falciparum*-positive samples from a previously conducted cross-sectional study in Boa Vista and Pacaraima (2). Sample selection prioritized cases with higher parasitemia levels to improve sequencing success and analysis quality. The parasitemia levels ranged from 3,420 to 108,600 parasites/μL, with a geometric mean of 11,097 parasites/μL (95% CI: 9,275–13,276), indicating that most infections were of moderate to high density. The selected samples comprised 38 cases from 2016 to 2017, 50 from 2018 to 2019, and 11 from 2020.

Eligibility and Sample Processing

Eligible participants were symptomatic, aged 16 years or older, and not pregnant or presenting signs of severe malaria. Peripheral blood samples were collected in EDTA tubes and stored at 4°C until

DNA extraction, which was performed using the QIAamp DNA Mini Kit (QIAGEN, Chatsworth, CA, USA). Initial diagnosis was performed by trained microscopists using Giemsa-stained thick blood smears, and *P. falciparum* infection was confirmed by qPCR as previously described (1, 3).

Recurrence Classification

Information on previous malaria episodes for each participant was obtained through Brazil's Malaria Epidemiological Surveillance System (SIVEP-Malaria), maintained by the Ministry of Health. Recurrence was defined as any new episode confirmed by microscopy occurring from day 3 to day 42 after the initial episode. This definition aligns with national clinical guidelines for therapeutic efficacy monitoring (4), which considers episodes within this time frame as potential late treatment failures. Since SIVEP-Malaria records are limited to symptomatic cases that prompted individuals to seek medical care, all recurrences identified during this study period were clinically apparent. Episodes occurring prior to the participant's formal enrollment in the study were also considered recurrences if they fell within the defined time interval.

Detection of *pfmdr1* and *pfcrt* SNPs

Genotyping of *pfmdr1* and *pfcrt* SNPs followed the protocol described by Fançony et al. (5). To detect *pfcrt* mutations (K76T, N326S, I356T), we employed PCR–restriction fragment length polymorphism (RFLP). Amplification products were purified using ExoSAP-IT™ (ThermoFisher), and sequencing reactions were performed with the BigDye™ Terminator v3.1 Cycle Sequencing Kit (Applied Biosystems). Capillary electrophoresis was carried out on an ABI 3730 PRISM® DNA Analyzer at the KI Gene Core Facility. The *pfcrt*-C350R mutation was detected using conventional PCR with Q5® Hot Start High-Fidelity DNA Polymerase (New England Biolabs), following the method by Florimond et al. (6). Chromas software was used for base-calling and visualization of DNA sequence chromatograms, while BioEdit was used for sequence alignment and editing.

Library preparation and Next-Generation Sequencing (NGS)

Libraries were prepared using the Magnis® NGS Prep System (Agilent Technologies), an automated platform optimized for high-efficiency target enrichment and consistent library quality. For each sample, 200 ng of *P. falciparum* genomic DNA was used in a 14 µL reaction. Target enrichment was achieved using a custom hybridization panel specific to *P. falciparum* genomic regions of interest. The library preparation workflow included pre-capture (7 cycles) and post-capture (12 cycles) amplification steps. Library quality and fragment size distribution were assessed with the Agilent TapeStation® system. Indexed libraries were pooled (10 nM final concentration) and sequenced on the Illumina NextSeq® 2000 platform at the National Genomics Infrastructure (NGI) at the SciLifeLab (Solna, Sweden).

Quality Control and Preprocessing of Sequencing Reads

Initial quality assessment of raw sequencing reads was performed using FastQC (v0.11.9), and results were aggregated into a consolidated summary report using MultiQC. Adapter trimming and removal of low-quality bases were conducted using Fastp (v0.23.4). To account for unique molecular identifiers (UMIs), the first and last six bases of each read were trimmed to reduce sequencing artefacts and improve accuracy in variant detection. Additional preprocessing steps included converting raw FASTQ files into unaligned BAM format using FastqToSam (Picard v3.2.0). Adapters were marked using MarkIlluminaAdapters, and the data were converted back to FASTQ format using SamToFastq, ensuring accurate handling of UMIs and removal of residual adapter sequences before alignment.

Read Alignment and Variant Detection

Cleaned sequencing reads were aligned to the *P. falciparum* reference genome (Pf3D7) using the BWA-MEM algorithm (v0.7.18). The genome was indexed in advance with BWA-Index to optimize alignment performance. BAM files were sorted and duplicates marked with MarkDuplicates (GATK v4.5.0.0). Variant calling was performed using Mutect2 (GATK v4.5.0.0), a sensitive algorithm

suitable for detecting low-frequency variants, including those from mixed infections or minor clones. Variants were filtered using FilterMutectCalls, and high-confidence variants were retained using SelectVariants (GATK v4.5.0.0). Final variant call format (VCF) files were further processed using bcftools (v1.20) for normalization and standardization. All the steps, from quality control to processing of the final VCFs, were orchestrated by an *in-house* pipeline developed in NextFlow (https://github.com/AntonioRezende/malvariant/tree/master).

Variant Annotation and Coverage Analysis

Identified variants were annotated using SnpEff, which classifies mutations based on predicted functional impact and genomic location. To assess sequencing depth in resistance-associated genes, coverage analysis was conducted using BEDTools (version not available) with a custom BED file. Coverage output was processed using a custom Python script that aggregated depth values, computed descriptive statistics (mean and standard deviation), and generated visual summaries using Matplotlib (v3.10.3) and Seaborn (v0.13.2). Python script is available in: https://github.com/yankaslzr/ngs_qc

REFERENCES

1. Abdallah R, Louzada J, Carlson C, Ljolje D, Udhayakumar V, Ferreira JO, Lucchi NW. 2022. Cross-border malaria in the triple border region between Brazil, Venezuela and Guyana. Sci Rep 12:1–8.

2. Louzada J, de Almeida NCV, de Araujo JLP, Silva J, Carvalho TM, Escalante AA, Oliveira-Ferreira J. 2020. The impact of imported Malaria by gold miners in roraima: Characterizing the spatial dynamics of autochthonous and imported malaria in an urban region of boa vista. Mem Inst Oswaldo Cruz 115:1–10.

3. Costa GL, Mascarenhas MEP, Martin TOG, Fortini LG, Louzada J, Pereira DB, Aguiar ACC, Carvalho LH, de Brito CFA, Fontes CJF, de Sousa TN. 2021. A Comprehensive Analysis of

the Genetic Diversity of Plasmodium falciparum Histidine-Rich Protein 2 (PfHRP2) in the Brazilian Amazon. Front Cell Infect Microbiol 11:742681.

4. World Health Organization. 2024. WHO guidelines for malaria. World Health Organization, Geneza.

5. Fançony C, Fortes-Gabriel E, Zage F, Alexiou E, Broumou I, Pernaute-Lau L, Panzo J, António EJ, Cristovão MS, Domingos JM, Sassoma E, Kuatoko F, Rosario EVN, Martins A, Färnert A, Bernardino L, de Sousa TN, Gil JP. 2025. Artemether-Lumefantrine Treatment Selects *Plasmodium falciparum* Multidrug Resistance 1 (*pfmdr1*) Increased Copy Number Among African Malaria Infections. J Infect Dis 231:1–10.

6. Florimond C, de Laval F, Early AM, Sauthier S, Lazrek Y, Pelleau S, Monteiro WM, Agranier M, Taudon N, Morin F, Magris M, Lacerda MVG, Viana GMR, Herrera S, Adhin MR, Ferreira MU, Woodrow CJ, Awab GR, Cox H, Ade MP, Mosnier E, Djossou F, Neafsey DE, Ringwald P, Musset L. 2024. Impact of piperaquine resistance in Plasmodium falciparum on malaria treatment effectiveness in The Guianas: a descriptive epidemiological study. Lancet Infect Dis 24:161–171.
